# The Reliability and Quality of YouTube Videos as a Source of Public Health Information Regarding COVID-19 Vaccination: Cross-sectional Study

**DOI:** 10.2196/29942

**Published:** 2021-07-08

**Authors:** Calvin Chan, Viknesh Sounderajah, Elisabeth Daniels, Amish Acharya, Jonathan Clarke, Seema Yalamanchili, Pasha Normahani, Sheraz Markar, Hutan Ashrafian, Ara Darzi

**Affiliations:** 1 Department of Surgery & Cancer Imperial College London London United Kingdom; 2 Institute of Global Health Innovation Imperial College London London United Kingdom; 3 Department of Mathematics Imperial College London London United Kingdom

**Keywords:** COVID-19, infodemiology, public health, quality, reliability, social media, vaccination, vaccine, video, web-based health information, YouTube

## Abstract

**Background:**

Recent emergency authorization and rollout of COVID-19 vaccines by regulatory bodies has generated global attention. As the most popular video-sharing platform globally, YouTube is a potent medium for the dissemination of key public health information. Understanding the nature of available content regarding COVID-19 vaccination on this widely used platform is of substantial public health interest.

**Objective:**

This study aimed to evaluate the reliability and quality of information on COVID-19 vaccination in YouTube videos.

**Methods:**

In this cross-sectional study, the phrases “coronavirus vaccine” and “COVID-19 vaccine” were searched on the UK version of YouTube on December 10, 2020. The 200 most viewed videos of each search were extracted and screened for relevance and English language. Video content and characteristics were extracted and independently rated against Health on the Net Foundation Code of Conduct and DISCERN quality criteria for consumer health information by 2 authors.

**Results:**

Forty-eight videos, with a combined total view count of 30,100,561, were included in the analysis. Topics addressed comprised the following: vaccine science (n=18, 58%), vaccine trials (n=28, 58%), side effects (n=23, 48%), efficacy (n=17, 35%), and manufacturing (n=8, 17%). Ten (21%) videos encouraged continued public health measures. Only 2 (4.2%) videos made nonfactual claims. The content of 47 (98%) videos was scored to have low (n=27, 56%) or moderate (n=20, 42%) adherence to Health on the Net Foundation Code of Conduct principles. Median overall DISCERN score per channel type ranged from 40.3 (IQR 34.8-47.0) to 64.3 (IQR 58.5-66.3). Educational channels produced by both medical and nonmedical professionals achieved significantly higher DISCERN scores than those of other categories. The highest median DISCERN scores were achieved by educational videos produced by medical professionals (64.3, IQR 58.5-66.3) and the lowest median scores by independent users (18, IQR 18-20).

**Conclusions:**

The overall quality and reliability of information on COVID-19 vaccines on YouTube remains poor. Videos produced by educational channels, especially by medical professionals, were higher in quality and reliability than those produced by other sources, including health-related organizations. Collaboration between health-related organizations and established medical and educational YouTube content producers provides an opportunity for the dissemination of high-quality information on COVID-19 vaccination. Such collaboration holds potential as a rapidly implementable public health intervention aiming to engage a wide audience and increase public vaccination awareness and knowledge.

## Introduction

The recent emergency authorization and rollout of COVID-19 vaccines by regulatory bodies has generated global media attention. Unsurprisingly, internet searches related to COVID-19 vaccination increased drastically during November-December 2020, as the public attempted to source information amid a surge in media coverage [1]. Many internet users turned to YouTube, the second-most visited website globally after Google, for further information [2].

YouTube is the most popular video-sharing platform worldwide. Over 1 billion hours’ worth of video is streamed each day on the website, and it is visited by over 2 billion unique users monthly [3]. It has strong penetrance globally and across all major sociodemographic groups. YouTube provides a potent means of disseminating real-time information across a population; users are able to curate video content from sources varying from individual users, through celebrities, to media outlets. Aware of their central role in the dissemination of key public health information, YouTube has implemented a COVID-19 medical misinformation policy, which forbids COVID-19–related content that contradicts local health authorities and risks public safety [4].

There have been, however, high-profile instances of internet-propagated misinformation regarding COVID-19, including the ingestion of cleaning products as potential treatment, which have had severe consequences [5]. Despite the aforementioned measures implemented by YouTube, there remains a concern for COVID-19 vaccination programs to remain an easy target for misinformation content. Previous studies have highlighted that vaccination programs, such as the human papillomavirus vaccination program, have been a common target of high-profile YouTube videos propagated by a community of vocal users who are critical of vaccination programs [6]. Furthermore, there remains a concern that anti–COVID-19 vaccination videos could (1) pose a significant threat to compliance with the vaccination program, especially among those who are disproportionately affected by the illness; (2) create spill-over dissonance toward other critical COVID-19 public health measures; and (3) displace notifications regarding other emerging time-sensitive information from official public health sources. In fact, from the beginning of the pandemic until now, COVID-19 vaccine hesitancy has been steadily increasing [7]. Understanding the nature of available content regarding the COVID-19 vaccination program on this widely used platform is, therefore, of substantial public health interest and forms a foundation on which strategies for misinformation counteraction can be based.

To date, no studies have evaluated the quality and reliability of COVID-19 vaccination–related information available on YouTube. The objective of this study, therefore, was to evaluate the reliability and quality of information of YouTube’s most prominent videos on COVID-19 vaccination, using 2 validated criteria (DISCERN and HONcode).

## Methods

### Methods Overview

Ethical approval for this study was waived because all gathered data are freely available in the public domain. The phrases “coronavirus vaccine” and “COVID-19 vaccine” were searched on the UK version of YouTube on December 10, 2020. The search was conducted in an incognito browser (Google Chrome) to avoid biased suggestions based on cookies. Search results were sorted by view count to identify videos that had achieved the greatest impact and were most likely to trend, thereby reaching further viewers. The 200 most viewed videos (10 pages) of each search were subsequently extracted.

Video titles and channels were first screened for relevance and English language before full-video screening. Videos were included if they described 1 or more of the following: mechanisms of action of vaccines, clinical trial procedures, manufacturing processes, side effects or safety, and vaccine efficacy. Descriptions of the criteria are provided in Multimedia Appendix 1, Table S1. If there was uncertainty regarding whether a video should be included, a consensus was sought between authors, with a predisposition to include the video for full assessment. Additionally, videos were assessed for their promotion of public health measures such as hand-washing, wearing masks, or social distancing. Finally, instances of nonfactual content in these videos were noted. Nonfactual information (ie, misinformation) was defined as nonscientifically corroborated content that contradicted medical information provided by the current local health authority or the World Health Organization. Examples of misinformation are available on YouTube’s medical misinformation policy [4]. Duplicate videos and non-English–language videos were excluded. Video content screening was completed independently by 2 authors (CC and ED). Any discrepancies were resolved through discussion with a third author (VS).

Characteristics (video URL, channel, country of origin, view count, duration, video age, and the number of likes, dislikes, and comments) of the included videos were extracted. Videos were placed in 6 main categories by YouTube channel type: educational channels produced by medical professionals, educational channels produced by nonmedical individuals (eg, science education or explanatory media), independent nonmedical users (eg, vloggers with no obvious affiliations), internet media (eg, newsmagazine shows or talk shows), news agencies (ie, clips uploaded from network news), and nonprofit or medical organizations (eg, hospitals, government organizations, or universities). Descriptions and examples of channel types are provided in Multimedia Appendix 1, Table S2.

Reliability of the video content (ie, the extent to which the source of information, and therefore the information itself, could be relied upon, evident from clearly referenced and scientifically corroborated content) was assessed against a modified Health on the Net Foundation Code of Conduct (HONcode) checklist [8] and modified DISCERN quality criteria for consumer health information [8,9], which have previously been used to assess the quality of health information on YouTube. The quality of video content (ie, completeness, understandability, relevance, depth, and accuracy of information provided) was also assessed using the DISCERN quality criteria. Video rating was completed independently by 2 authors (CC and ED).

HONcode consists of 8 principles that evaluate the reliability and credibility of health information [10]. Videos were rated with a score of 1 (adherent) or 0 (nonadherent) for each of the 8 principles. The DISCERN instrument consists of 16 questions rated from 1 to 5, which assess health content across 3 domains including video reliability (8 questions, 40 points), treatment information quality (7 questions, 35 points), and an overall reviewer rating (5 points), thus yielding a maximum cumulative score of 80 (Textbox 1) [11]. The first 8 questions were applied to all videos, and 8 additional questions were applied to videos specifically on vaccine science (ie, how the treatment works). The questionnaire was adapted from Goobie et al [8] and Loeb et al [9].

**Modified DISCERN quality criteria for assessing the reliability and quality of YouTube content on COVID-19 vaccination. Each question was rated from 1 (“worst”) to 5 (“best”). Sections 2 and 3 only applied to videos on vaccine science (ie, how the treatment [vaccination] works).**

**Section 1: is the video reliable?**

1. Are the aims clear?

2. Does it achieve its aims?

3. Is it relevant?

4. Is it clear what sources of information were used to compile the video?

5. Is it clear when the information used or reported in the video was produced?

6. Is it balanced and unbiased?

7. Does it provide details of additional sources of support and information?

8. Does it refer to areas of uncertainty?

**Section 2: how good is the quality of information on treatment choices?**

9. Does it describe how each treatment works?

10. Does it describe the benefits of each treatment?

11. Does it describe the risks of each treatment?

12. Does it describe what would happen if no treatment is used?

13. Does it describe how the treatment choices affect overall quality of life?

14. Is it clear that there may be more than one possible treatment choice?

15. Does it provide support for shared decision-making?

**Section 3: overall rating of the video**

16. Based on the answers to all of the above questions, rate the overall quality of the video as a source of information about treatment choices

### Statistical Analysis

Statistical analysis was performed using Stata (version 13, StataCorp). Intercategory differences were assessed using Kruskal–Wallis tests and the post hoc Dunn test. Interrater reliability was assessed using the Cohen κ statistic. A DISCERN score of +1 or –1 point was considered agreement. Associations between engagement metrics and DISCERN scores were evaluated using linear regression. Significance was set at *P*<.05. Data are presented as median (IQR) values.

### Availability of Data and Material

The data sets used or analyzed in this study are available from the corresponding author on reasonable request.

## Results

### Video Characteristics

**Figure 1:**
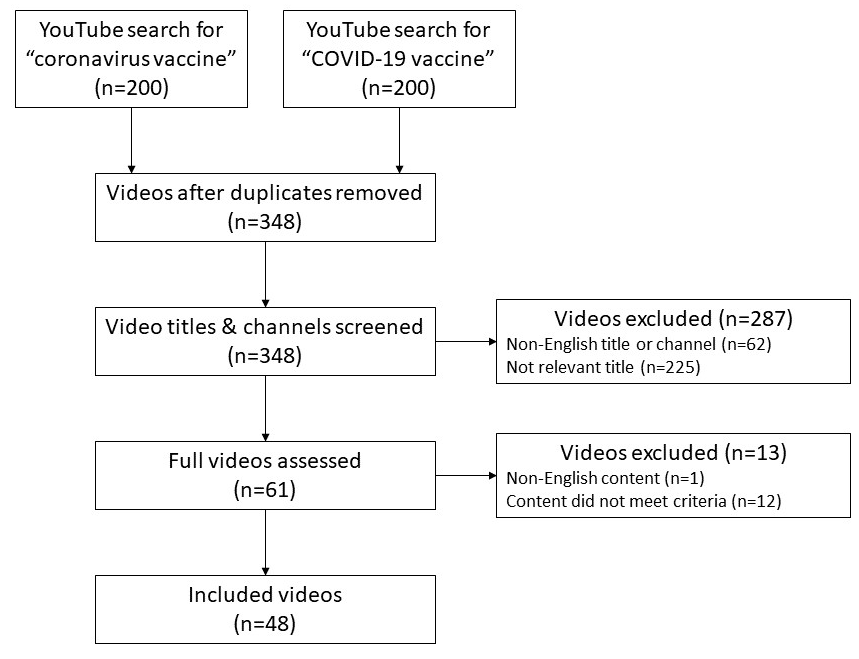
Flow diagram for the results for searches on COVID-19 vaccine–related videos on YouTube and the video selection process for inclusion in the study. The 2 searches were performed on December 10, 2020.

The video review process is illustrated in Figure 1. From the 200 results of each search, 52 duplicate videos were removed, yielding 348 unique videos. After the video title and channel were screened and the full video was assessed, 48 videos were included for data extraction, with a combined total view count of 30,100,561. Videos that were not in English (n=62) or did not meet the study inclusion criteria (n=225)—describing topics such as vaccination priority, national distribution plans, politics, or pandemic mortality figures—were excluded. The characteristics of the included videos are summarized in Table 1. The majority (75%) of videos were produced by US channels. The median number of views per video was 236,064 (IQR 152,082-596,234).

**Table 1:** Characteristics of COVID-19 vaccine–related YouTube videos included in the study.

Characteristics		Values
**Country of origin, n (%)**		
	United States	34 (75)
	United Kingdom	6 (13)
	Canada	3 (6)
	Germany	2 (4)
	Australia	1 (2)
	Switzerland	1 (2)
**Channel type, n (%)**		
	Educational (nonmedical)	10 (21)
	Educational (medical)	6 (13)
	Independent users	5 (10)
	Internet media	10 (21)
	News agencies	13 (27)
	Nonprofit or medical organizations	4 (8)
Video duration (minutes), median (IQR)		9:18 (4:42-11:51)
Video age (days since upload), median (IQR)		54 (19-191)
**Engagement, median (IQR)**		
	Views	236,064 (152,082-596,234)
	Views per day since upload	6364 (3031-11,717)
	Likes	5600 (2300-9200)
	Dislikes	545 (236-1200)
	Likes:dislikes ratio	14.3 (3-24)
	Comments	1891 (1059-3424)

### Source

The 48 videos were segregated into 6 categories by their YouTube channel type. The contribution of each channel type toward the total view count is detailed in Table 2. The most viewed video (6,668,737 views, 22% of total views) described the mechanisms of action of COVID-19 vaccines and was produced by a medical organization (JAMA Network).

**Table 2:** Engagement metrics, content, adherence to the Health on the Net Foundation Code of Conduct (HONCode), and DISCERN score of COVID-19 vaccine–related videos on YouTube, stratified by channel type.

Parameters		Educational (nonmedical)	Educational (medical)	Independent nonmedical users	Internet media	News agencies	Nonprofit or medical organizations	Overall	*P* value^a^	
Videos, n (%)		10 (21)	6 (13)	5 (10)	10 (21)	13 (27)	4 (8)	48 (100)	N/A^b^	
Total views, n (%)		5,473,987 (18)	1,467,003 (5)	1,766,051 (6)	4,205,972 (14)	9,798,419 (33)	7,389,129 (25)	30,100,561 (100)	N/A	
Views per video, median (IQR)		367,560 (162,635-623,924)	213,707 (143,570-302,387)	221,299 (209,150-334,553)	197,365 (135,953-557,721)	275,615 (137,721-644,970)	247,977 (241,877-1,853,382)	236,064 (152,082-596,234)	N/A	
Views per day since upload, median (IQR)		7604 (3681-10,802)	8309 (5179-9699)	8852 (7406-27,879)	1984 (880-5470)	5560 (4279-10,656)	11,734 (2532-37,832)	6364 (3031-11,717)	N/A	
Likes, median (IQR)		15,050 (6050-25,250)	5600 (5000-6725)	10,000 (5800-10,000)	4200 (2000-6275)	2600 (1300-5100)	3400 (1998-15,200)	5600 (2300-9200)	N/A	
Dislikes, median (IQR)		656 (261-1325)	268 (213-405)	557 (342-701)	651 (223-1200)	716 (404-1200)	1700 (970-5700)	545 (236-1200)	N/A	
Likes:dislikes ratio, median (IQR)		23.8 (18-26)	20.9 (18-25)	18 (6-30)	12.3 (3-18)	4.3 (2-11)	2.8 (2-8)	14.3 (3-24)	N/A	
Comments, median (IQR)		1633 (1047-3444)	1932 (1691-2253)	2133 (1680-6402)	1430 (622-3480)	2204 (1194-3611)	1258 (735-1858)	1891 (1059-3424)	N/A	
**Content^c^, n (%)**										N/A
	Vaccine science	9 (90)	6 (100)	1 (20)	5 (50)	4 (31)	3 (75)	28 (58)		
	Trial process	5 (50)	5 (83)	1 (20)	7 (70)	8 (62)	2 (50)	28 (58)		
	Manufacturing process	2 (20)	0 (0)	0 (0)	4 (40)	2 (15)	0 (0)	8 (17)		
	Side effects and safety	3 (30)	5 (83)	5 (100)	1 (10)	7 (54)	2 (50)	23 (48)		
	Vaccine efficacy	2 (20)	5 (83)	3 (60)	2 (20)	4 (31)	1 (25)	17 (35)		
	Public health information	3 (30)	1 (17)	1 (20)	3 (30)	1 (8)	1 (25)	10 (21)		
**HONcode adherence (/8), n (%)**										N/A
	Low (0-2)	2 (20)	1 (17)	5 (100)	5 (50)	10 (77)	3 (75)	27 (56)		
	Moderate (3-5)	7 (70)	5 (83)	0 (0)	4 (40)	3 (23)	1 (25)	20 (42)		
	High (6-8)	1 (10)	0 (0)	0 (0)	1 (10)	0 (0)	0 (0)	1 (2)		
**DISCERN score, median (IQR)**										
	Reliability (/40, n=48)	28.5 (25.9-32.5)	35.5 (32.0-39.4)	18 (18-20)	24.8 (23.3-26.8)	23.8 (20.5-24.5)	23.5 (22.9-24.5)	25 (22.9-29.1)	*<.001*	
	Treatment quality (/35, n=30)	16.5 (14-18)	21.3 (20.1-25.8)	19 (19-19)	17.5 (17.0-18.5)	14.8 (12.6-17.4)	20.5 (16.8-21.3)	18.3 (15.1-20.5)	.052	
	Overall quality judgement (/5, n=30)	3 (3-4)	4.8 (4.1-5.0)	3.5 (3.5-3.5)	3 (2.5-4.0)	2.5 (2.0-3.1)	3 (3.0-3.5)	3.3 (3-4)	.07	
	Total score (/80, n=30)	48.5 (46.5-53.0)	64.3 (58.5-66.3)	43.5 (43.5-43.5)	49 (45.5-52.0)	40.3 (34.8-47.0)	52 (45.5-54.3)	50 (45.1-53.1)	*.03*	

^a^*P* values were produced using Kruskal-Wallis tests. Significant figures are in italics.

^b^N/A: not applicable.

^c^Percentage values have been calculated relative to the total number of videos of each channel type: educational (nonmedical) (n=10), educational (medical) (n=6), independent nonmedical users (n=5), internet media (n=10), news agencies (n=13), and nonprofit or medical organizations (n=4).

### Content

Twenty-eight of 48 (58%) videos addressed vaccine science and mechanisms of action. Twenty-eight videos also discussed vaccine trials, and 23 videos discussed vaccine safety or side effects. Ten videos advocated the importance of continued traditional public health measures to reduce COVID-19 transmission (eg, hand-washing, mask-wearing, and social distancing).

Regarding nonfactual content, 2 videos (1 by internet media and 1 independently produced) contained unsubstantiated vaccine safety concerns, despite YouTube’s aforementioned COVID-19 misinformation policy. Both videos were interviews with a single prominent antivaccination advocate. These 2 nonfactual videos accounted for 390,927 views (1.3% of total viewership).

### Adherence With HONcode and DISCERN Principles

There was strong interrater agreement for both HONcode principles (median 94%, IQR 93%-97%; median κ=0.81, IQR 0.73-0.87) and DISCERN (median 88%, IQR 82-94%; median κ=0.83, IQR 0.76-0.91).

Forty-seven of 48 (98%) videos had either low (56%) or moderate (42%) adherence with HONcode principles. In general, videos scored poorly regarding the disclosure of financial sources and advertising (Table 3). Regarding the “authoritative” domain, only approximately half of the videos had an input from a medical professional or relevant scientist. Additionally, only a minority of videos fulfilled criteria relating to the “attribution,” “justifiability,” and “transparency” of the data presented.

**Table 3:** Description of principles of the Health on the Net Foundation Code of Conduct [8] and the number of COVID-19 vaccine–related YouTube videos that met each criterion.

Principle	Description	Videos, n (%)
Authoritative	Any medical or health advice provided in this video will only be given by medically trained and qualified professionals unless a clear statement is made that the advice offered is from a nonmedically qualified individual or organization.	27 (56)
Complementary	The information provided is designed to support, not replace, the relationship that exists between a patient and his/her existing physician.	38 (79)
Privacy	The information in the video maintains the right to confidentiality and respect of the individual patient featured.	0 (0)
Attribution	Where appropriate, information contained in the video will be supported by clear references to source data and, where possible, have specific links to those data.	20 (42)
Justifiability	Any claims relating to the benefits or performance of a specific treatment, commercial product, or service will be supported by appropriate, balanced evidence in the manner outlined in in attribution principle.	20 (42)
Transparency	The designers of the video will seek to provide information in the clearest possible manner and provide contact addresses for viewers who seek further information or support.	17 (35)
Financial disclosure	Support for this video will be clearly identified, including the identities of commercial and noncommercial organizations that have contributed funding, services, or material for the video.	7 (15)
Advertising policy	If advertising is a source of funding, it will be clearly stated. Advertising and other promotional material will be presented to viewers in a manner and context that facilitate differentiation between it and the original content.	2 (4)

There were significant differences in DISCERN reliability and overall scores among different channel types (*P*<.001 and *P*=.03, respectively). Educational channels produced by medical professionals attained the greatest median (IQR) DISCERN scores for reliability (median 35.5, IQR 32.0-39.4), quality (median 21.3, IQR 20.1-25.8), user judgement (median 4.8, IQR 4.1-5), and overall score (median 64.3, IQR 58.5-66.3). Videos produced by independent nonmedical individuals achieved the lowest reliability score (median 18, IQR 18-20). The post hoc Dunn test revealed that educational channels produced by medical professionals attained significantly higher overall DISCERN scores than nonmedical educational (*P*=.01), independent (*P*=.02), internet media (*P*=.01), and news channels (*P*<.001). Additionally, both educational channels produced by medical and nonmedical professionals achieved significantly higher DISCERN reliability scores than those produced by independent nonmedical users (*P*<.001 and *P*<.001, respectively), internet media (*P*=.007 and *P*=.04, respectively), and news channels (*P*<.001 and *P*=.003, respectively).

Regression analysis revealed no significant association between engagement metrics and DISCERN or HONcode scores (Multimedia Appendix 1, Table S3). However, there was a significant positive association between DISCERN and HONcode ratings for all videos included in the study (*P*<.001; *r*^2^=.583).

## Discussion

### Principal Findings

This study highlights the importance of YouTube as a medium for sharing of curated COVID-19–related information. We demonstrate growing public interest in extracting vaccine-related content from this resource, with the videos shortlisted in this study having been viewed over 30 million times globally thus far, and an average of 1890 comments in the discussion thread of each video. The available content appears favorably received, with a mean of 14.3 likes per dislike per video. This study, however, demonstrates the varying quality of information provided on YouTube, with 98% of reviewed content with low to moderate adherence to HONcode principles and DISCERN reliability scores ranging from 18 (nonmedical individuals) to 35.5 (educational channels) out of 40.

Despite variable video quality, our search identified only 2 (4.2%) videos that would constitute mis- or disinformation, which accounted for only 1.3% of the total viewership. In comparison, studies evaluating misinformation on YouTube, published in March and June 2020, highlighted a significantly higher proportion of videos containing misleading or nonfactual information [12,13]. This is probably owing to YouTube’s COVID-19 medical misinformation policy, which came into effect on October 14, 2020. YouTube now operates a “three strikes” system to prevent users from uploading unsubstantiated videos, which parallels the COVID-19 misinformation policies of Facebook and Twitter [14,15]. While the policy is explicitly directed at videos that contain unreal claims, such as the COVID-19 vaccines “kill people who receive them” or “contain a microchip,” tackling more insidious forms of misinformation in a timely manner has proven difficult. Recent criticism of these policies has highlighted their reliance on scientific consensus from health authorities to determine what exactly constitutes misinformation [16]. In such a rapidly developing field, with limited longitudinal evidence, this consensus cannot be readily achieved, which allows time for inaccurate social media content to be shared. This is of particular concern since previous studies on vaccine hesitancy have reported that videos of a negative tone are more likely to be shared and liked, thus perpetuating misinformation and confirmation biases [17]. In an effort to rapidly combat this, there has been increased government engagement and centralization of initiatives to reduce and prevent the spread of misinformation. The World Health Organization, in partnership with government agencies, has introduced several initiatives to improve public awareness of and to tackle vaccine-related misinformation (the so-called “infodemic”) on the internet [18,19]. Additionally, social media companies and the government of the United Kingdom have agreed on a battery of measures to reduce vaccine disinformation through rapid removal of flagged content and increased cooperation with public health bodies to ensure that authoritative messages regarding vaccine safety are disseminated to as many individuals as possible [20].

Along with user-directed policies, several other strategies have been suggested to limit the dissemination of false health information on social media platforms. These include mobilizing medical professionals as advocates to counter the propagation of misinformation [21]. Among the videos reviewed in our study, less than one-third were posted by nonprofit or medical organizations or medical professionals, which accounts for a lower proportion than that posted by news agencies. Furthermore, videos from established health-related organizations such as JAMA Network or World Health Organization only accounted for 25% of the total viewership. While there has been an exponential growth in medical YouTubers, and despite the fact that videos produced by these individuals achieved the highest reliability and quality scores, concurrent with other studies, we found that their current role remains limited [22]. Of note, the most viewed shortlisted video was developed by JAMA Network, which may suggest the importance of brand recognition or marketing in attracting audiences.

In addition to the varied provenance of available vaccine-related content, we identified a paucity of reliable information on YouTube. Information in videos produced by reputable health-related organizations was significantly more reliable than that obtained from videos produced by only nonmedical individual users (*P*=.007), and its reliability was comparable to videos from all other categories. The majority also only achieved “low adherence” to HONcode principles. Even though most of the videos produced by these nonprofit or medical organizations explained vaccine concepts in a clear and approachable manner, often utilizing the “infographic” format, they did not cite sources or provide links to further information, a common phenomenon in videos produced by established educational channels on YouTube. Thus, these videos were unable to fulfil both DISCERN reliability indicators (eg, “referencing of information” and “directing viewers to additional sources of knowledge”) and HONcode principles (eg, attribution and transparency) and were unable to attain high scores.

While nonfactual claims were limited to a small minority of videos, the absence of key information—particularly regarding basic vaccinology and the importance of concurrent public health measures—currently limits the utility of YouTube videos as robust sources of public health information. These findings echo those of previous studies reporting that reliability and quality of non–vaccine-related information on COVID-19 on YouTube is unsatisfactory [13,23]. As such, viewers are provided with incomplete evidence as to how the COVID-19 vaccine fits into the larger public health effort and are not provided with curated resources that could potentially provide these pertinent details.

### Limitations and Future Directions

There are some limitations to this study. First, the subset of videos examined were limited to the English language only, given that 88% of the videos were from the YouTube channels from the United States or the United Kingdom. While this represents a language bias and limits the generalizability of our findings to different languages and non–English-speaking countries, we note that similar findings have been reported with respect to COVID-19–related information in other languages [13].

Second, the search strategy was limited to 2 search phrases (“coronavirus vaccine” and “COVID-19 vaccine”). These phrases would not encompass the various searches the general public may make on this topic (eg, “covid vaccine,” “coronavirus vaccination,” or “covid vaccination”), which could yield different video results. Additionally, the search terms used were “neutral” and may not reflect searches made by individuals who (1) have already been previously subjected to misinformation, (2) have a network that shares similar misinformation content, or (3) are part of groups that are more likely to seek misinformation.

Third, videos were first screened by title relevance and for the purpose of pragmatism, only those videos that had titles relevant to 5 domains of COVID-19 vaccine information (mechanisms of action of vaccines, clinical trial procedures, manufacturing processes, side effects and safety, and vaccine efficacy) were considered for full video analysis. However, videos with nonrelevant titles may still contain relevant information on COVID-19 vaccination and given that they are accessible by the public through neutral search terms, they could contribute toward the dissemination of incorrect or low-quality information. Additionally, a large majority of search results were excluded at the screening stage, which resulted in a relatively small sample size. This process may have introduced a selection bias, limiting the generalizability of our findings. For completeness of reporting in the future, all videos in the search results should be analyzed for low-quality or incorrect information.

Fourth, the search was conducted at a single timepoint (December 10, 2020), which was relatively early in the timeline of global COVID-19 vaccine distribution. Given the dynamic nature of the COVID-19 pandemic and vaccine development, knowledge and attitudes regarding vaccination may evolve with increased scientific understanding and public health interventions. Moreover, the search results used in this study led to the inclusion of videos produced in late March and April when vaccine development was still in its early stages. Therefore, topics such as vaccine manufacturing, efficacy, or safety were not discussed in this early sample. These videos instead concentrated on the explanation of vaccine science and the methodology of clinical trials. A cross-sectional analysis of YouTube videos through searches at multiple timepoints or of those stratified by the age of the video could be conducted in the future to assess the progression of video content and quality.

Fifth, although used in previous studies that assessed YouTube as a source of medical information, DISCERN and HONcode principles were developed and validated for the assessment of written medical information. However, a strong interrater agreement between the scoring systems suggests they are reasonable tools to use in the absence of a validated alternative.

Finally, although efforts were made to reduce selection bias by performing the search in an incognito window, the physical search location could still be revealed to YouTube through the IP address. As such, further studies should consider assessing the nature of content that users are exposed to at different locations, perhaps carrying out stratifying analysis using socioeconomic markers such as index of multiple deprivation. Additionally, routes of misinformation may vary depending on culture, education level, and even at a national level. Social and ethnic determinants have been demonstrated to impact vaccine hesitancy [24,25]. It is important to understand the drivers of vaccine hesitancy and develop high-quality, widely available educational resources to target these demographic groups and improve vaccine uptake. Creating videos in collaboration with medical professionals and taking advantage of YouTube’s widespread reach represents one potential solution.

### Conclusions

Our findings demonstrate that YouTube videos produced by educational channels, especially those produced by medical professionals, achieve the highest quality and reliability metrics. Consistent with previous similar studies, this suggests that there is currently a missed opportunity in collaboration between respected health-related organizations and established educational YouTube content producers to disseminate high-quality information on COVID-19 vaccination [26]. This could potentially be a rapidly implementable public health intervention to engage a wider audience and increase public awareness and knowledge.

## Appendix

Multimedia Appendix 1 Supplementary tables.

## Abbreviations

HONcode: Health on the Net Foundation Code of Conduct
