# Supplementary material for: The Reliability and Quality of YouTube Videos as a Source of Public Health Information Regarding COVID-19 Vaccination: Cross-sectional Study
Source: JMIR Public Health Surveill. 2021 Jul 8;7(7):e29942. doi: 10.2196/29942 (PMC8274673; doi:10.2196/29942)
Supplement: Multimedia Appendix 1 [file publichealth_v7i7e29942_app1.docx]

**Table S1. Content criteria for inclusion of COVID-19 vaccine YouTube videos in the study. Any video must explain at least one descriptor of the first five categories to be included.**

| **Category** | **Description** |
| --- | --- |
| Vaccine basic science | Vaccine definition  Types of vaccines  Differences between types of vaccines  mRNA vaccine contents  mRNA vaccine mechanisms of action  Comparisons of different COVID-19 vaccines |
| Trial process | Stages of clinical trials  Purpose of clinical trials  Differences in human/animal trials  Challenge studies  Participant enrolment  Compressed timeline of clinical trials  Progress of current vaccine clinical trials  Clinical trial endpoints |
| Manufacturing process | Vaccine production processes  Cost of vaccine development |
| Vaccine efficacy | Efficacy definition  Efficacies of different vaccines |
| Side effects/safety | Definition of adverse events  Vaccine adverse events |
| **Also screening for:** |  |
| Public health information | Support for existing health measures such as handwashing, wearing face masks or social distancing |

**Table S2. Descriptions and examples of the YouTube channel categories in the study.**

| **Category** | **Description** | **Example channels** |
| --- | --- | --- |
| Educational (non-medical) | Educational channels produced by non-medical professionals that aim to improve science or health understanding. | AsapSCIENCE, SciShow, It’s Okay To Be Smart |
| Educational (medical) | Educational channels produced by medical professionals that aim to improve health understanding. | MedCram, Dr John Campbell, Medlife Crisis |
| Independent users | Individuals with no professional credentials or established affiliations such as vloggers. | PowerfulJRE, Russell Brand, vlogbrothers |
| Internet media | Channels produced by media organisations that produce entertainment media | 700 Club Interactive, Seeker, WIRED |
| News agencies | Clips from network news or uploaded by reputable newspapers | CNN, CBS, BBC News |
| Non-profit/medical organisations | Channels produced by non-profit or medical organisations such as universities, governmental agencies and hospitals | Cambridge University, JAMA Network, World Health Organisation |

**Table S3. Associations between engagement metrics and DISCERN and HONcode quality/reliability ratings.**

|  | **Co-efficient** | **95% CI** | ***P*-value** | **R^2^** |
| --- | --- | --- | --- | --- |
| **HONcode total score** |  |  |  |  |
| Total views | -1.07x10^-7^ | -5.22x10^-7^ – 3.08x10^-7^ | .606 | .0058 |
| Views per day | 3.98x10^-6^ | 1.1x10^-5^ – 1.9x10^-5^ | .596 | .0062 |
| Total likes | 4.63x10^-6^ | 2.78x10^-5^- 3.71x10^-5^ | .775 | .0018 |
| Total dislikes | 4.9x10^-5^ | -1.08x10^-4^ – 2.06x10^-4^ | .533 | .0087 |
| Like-dislike ratio | 0.0153 | -0.0188 – 0.0494 | .370 | .0179 |
| Total comments | -8.26x10^-4^ | -2.70x10^-4^ – 1.04x10^-4^ | .378 | .0177 |
| **DISCERN treatment quality** |  |  |  |  |
| Total views | 2.88x10^-7^ | -1.25x10^-6^ – 1.82x10^-6^ | .708 | .0031 |
| Views per day | 6.32x10^-6^ | -4.94x10^-5^ – 6.21x10^-5^ | .821 | .0011 |
| Total likes | 2.94x10^-5^ | -9.04x10^-5^ – 1.49x10^-4^ | .623 | .0054 |
| Total dislikes | -5.5x10^-5^ | -6.39x10^-4^ – 5.30x10^-4^ | .851 | .0008 |
| Like-dislike ratio | 0.684 | -0.0573 – 0.194 | .279 | .0260 |
| Total comments | -3.85x10^-4^ | -1.09x10^-3^ – 3.17x10^-4^ | .275 | .0270 |
| **DISCERN treatment quality** |  |  |  |  |
| Total views | -6.26x10^-9^ | -1.33x10^-6^ – 1.32x10^-6^ | .992 | <.0001 |
| Views per day | -9.53x10^-7^ | -6.63x10^-5^ – 6.43x10^-5^ | .976 | >.0001 |
| Total likes | -2.31x10^-5^ | -1.33x10^-4^ – 8.68x10^-5^ | .668 | .0075 |
| Total dislikes | 2.85x10^-5^ | -4.35x10^-4^ – 4.92x10^-4^ | .900 | .0006 |
| Like-dislike ratio | 0.0961 | -0.0316 – 0.224 | .134 | .0876 |
| Total comments | 7.18x10^-5^ | -9.38x10^-4^ – 1.08x10^- 3^ | .885 | .0009 |
| **DISCERN overall quality** |  |  |  |  |
| Total views | 1.65x10^-7^ | -2.71x10^-6^ – 3.04x10^-^6 | .907 | .0005 |
| Views per day | 4.47x10^-5^ | -9.55x10^-5^ – 1.85x10^-4^ | .518 | .0162 |
| Total likes | -3.39x10^-5^ | -2.76x10^-4^ – 2.08x10^-4^ | .775 | .0033 |
| Total dislikes | -7.01x10^-5^ | -1.09x10^-3^ – 9.47x10^-4^ | .888 | .0008 |
| Like-dislike ratio | 0.216 | -0.0635 – 0.496 | .124 | .0921 |
| Total comments | 4.82x10^-4^ | -1.62x10^-3^ – 2.60x10^-3^ | .643 | .0091 |
